# Did HealthKick, a randomised controlled trial primary school nutrition intervention improve dietary quality of children in low-income settings in South Africa?

**DOI:** 10.1186/s12889-015-2282-4

**Published:** 2015-09-23

**Authors:** Nelia P. Steyn, Anniza de Villiers, Nomonde Gwebushe, Catherine E. Draper, Jillian Hill, Marina de Waal, Lucinda Dalais, Zulfa Abrahams, Carl Lombard, Estelle V. Lambert

**Affiliations:** Division Human Nutrition, University of Cape Town, P/Bag X3, Observatory, Cape Town, 7925 South Africa; Non-communicable Diseases Research Unit, South African Medical Research Council, Francie van Zijl Drive, Tygerberg, Cape Town, 7505 South Africa; Biostatistics Unit, South African Medical Research Council, Francie van Zyl Drive, Tygerberg, Cape Town, 7505 South Africa; Division of Exercise Science and Sports Medicine, Department of Human Biology, Faculty of Health Sciences, University of Cape Town, Cape Town, 8001 South Africa; Division Nutrition, University of Cape Town, Cape Town, 8001 South Africa; BroadReach Healthcare, Park Lane Office Park, cnr Alexandra Road & Park Lane Pinelands, Cape Town, 7405 South Africa; Division of Exercise Science and Sports Medicine, Sports Science Institute of South Africa, Boundary Road, Newlands, 8001 Cape Town, South Africa

**Keywords:** Schools, Nutrition, Intervention, HealthKick, South Africa

## Abstract

**Background:**

Numerous studies in schools in the Western Cape Province, South Africa have shown that children have an unhealthy diet with poor diversity and which is high in sugar and fat. HealthKick (HK) was a three-year randomised controlled trial aimed at promoting healthy eating habits.

**Methods:**

Sixteen schools were selected from two low-income school districts and randomly allocated to intervention (*n* = 8) or control school (*n* = 8) status. The HK intervention comprised numerous activities to improve the school nutrition environment such as making healthier food choices available and providing nutrition education support. Dietary intake was measured by using a 24-h recall in 2009 in 500 grade 4 learners at intervention schools and 498 at control schools, and repeated in 2010 and 2011. A dietary diversity score (DDS) was calculated from nine food groups and frequency of snack food consumption was determined. A school level analysis was performed.

**Results:**

The mean baseline (2009) DDS was low in both arms 4.55 (SD = 1.29) and 4.54 (1.22) in the intervention and control arms respectively, and 49 % of learners in HK intervention schools had a DDS ≤4 (=low diversity). A small increase in DDS was observed in both arms by 2011: mean score 4.91 (1.17) and 4.83 (1.29) in the intervention and control arms respectively. The estimated DSS intervention effect over the two years was not significant [0 .04 (95 % CI: −0.37 to 0.46)]. Food groups least consumed were eggs, fruit and vegetables. The most commonly eaten snacking items in 2009 were table sugar in beverages and/or cereals (80.5 %); followed by potato crisps (53.1 %); non-carbonated beverages (42.9 %); sweets (26.7 %) and sugar-sweetened carbonated beverages (16 %). Unhealthy snack consumption in terms of frequency of snack items consumed did not improve significantly in intervention or control schools.

**Discussion:**

The results of the HK intervention were disappointing in terms of improvement in DDS and a decrease in unhealthy snacking. We attribute this to the finding that the intervention model used by the researchers may not have been the ideal one to use in a setting where many children came from low-income homes and educators have to deal with daily problems associated with poverty.

**Conclusions:**

The HK intervention did not significantly improve quality of diet of children.

## Background

Numerous studies in South Africa have shown that children and adolescents eat a range of unhealthy snacks, particularly at school, and frequently in low-income low-income settings [1–3]. Generally these snacks tend to be high in saturated fat, total fats, sugar, and salt and low in fibre and micronutrients. The diet tends to be very energy-dense.

Diets of this nature have been well documented to lead to the development of obesity and ultimately to certain non-communicable diseases (NCDs) in adulthood [4, 5]. Since South Africa has a rising prevalence of obesity and many associated NCDs [6, 7] it is prudent to introduce preventative measures as early as possible in childhood. Hence, schools become important vehicles for teaching children about a healthy diet and physical activity, since children spend a large part of their time at school and are readily accessible to health promotion strategies [8].

One way of fostering nutrient adequacy in the diet is by promoting one having as much diversity as possible [9]. A diet which is low in diversity is associated with micronutrient deficiencies [9, 10], growth stunting [11], cardiovascular risk [12], and dyslipidaemia [13]. High dietary diversity (nutrition wellness) is generally associated with consumption from as many different food groups as possible [14]. Additionally, a healthy diet for children should be limited in total fat, saturated fat, added sugar, and salt and contain at least five portions fruit/vegetables per day [15].

The HealthKick (HK) intervention was initiated in primary schools in low-income settings in the Western Cape (WC) Province. This was the first intervention study in South Africa that aimed to promote healthy eating habits and regular physical activity in learners, parents and educators by means of an action planning process. Furthermore, the programme aimed to promote the development of a school environment which would foster a healthy lifestyle. One of the primary dietary strategies associated with the nutrition aspects of the intervention was to promote dietary diversity and the nutrition education messages promoted were: *“Eat a variety of foods every day”* [16] and *“Eat many different types of fruit and vegetables every day”* [17] since these are two of the South African food-based dietary guidelines aimed at improving the diet of all South Africans [18]. Additionally, a low fat and low sugar intake were promoted by using the following messages which were taken from the food-based dietary guidelines, namely: *“Eat less fatty and oily food*” [19] and “*Eat less sugar and sweet foods, such as cakes, doughnuts and sweets”* [20]. Hence the research question was: “Can a low impact school nutrition intervention programme improve the quality of the diet of primary school children?” and the aim of the present study was to evaluate the dietary outcomes of the HK intervention regarding dietary variety, sugar, and fat intake over a period of three years.

## Methods

### Participants

Sixteen schools were purposefully selected from two school districts in the WC. Schools were paired with a school that had approximately the same; ethnic, language and socio-economic profile. The schools were from the lowest three socio-economic quintiles. These quintiles take into account weighted household data on income dependency ratio (or unemployment rate), and the level of education of the community (or literacy rate) as reflected in national census data. Quintile one (Q1) is the poorest quintile and quintile five (Q5) the least poor. Schools within a pair were then randomly assigned to either the intervention (*n* = 8) or control arm (*n* = 8) of the study. In 2009, 500 learners in the eight intervention and 498 in the control schools were randomly selected for the dietary intake questionnaire from those learners in grade 4 classes whose parents gave consent for their children to participate in the survey. This process was repeated in 2010 and 2011 for learners from grades 5 and 6 classes.

### The HealthKick intervention

The HK programme was planned within the context of the socio-ecological model and comprised three phases which have been described in some detail elsewhere [21]. Briefly, intervention mapping, followed by a formative assessment in 100 schools in the WC, was first undertaken to identify objectives and priorities for the intervention [22]. This was followed by a baseline study in eight intervention and eight control schools to collect basic data (socio-economic, diet, physical activity, health, and knowledge) from learners, educators, parents, and the school environment. The third phase comprised the action planning component of the HK intervention which took place over the period of a year. This process was based on Action Schools! BC (AS! BC) [23–26] intervention model for Schools and Teachers, and the Centres for Disease Control School Health Index [27, 28] which is a self-assessment and planning guide.

The AS! BC model is classified as a comprehensive whole school health model [23–26]. It provides tools for schools and educators to create individualised “ACTION PLANS” that increase opportunities for physical activity and healthy eating across four action zones. The educators were provided with training and resources required to implement their action plans. Educators were asked to give an additional 15 min of physical activity a day and at least one healthy eating activity per month.

In the present study the aim was to guide the ‘champion’, principal, and staff at the intervention schools through a process which enabled them to assess areas for actions related to nutrition and physical activity, identify priorities and set feasible goals. In other words, the schools set their own goals. Over the three years the schools planned and implemented the activities they had identified. These included (among other things) nutrition activities related to developing healthy school nutrition policies; providing a nutrition education support; improving school shops by making healthier options available; encouraging the promotion of healthy foods at special events; by encouraging learners to bring healthy lunch boxes to school; and by the initiation of vegetable gardens at schools. This process has been described in greater detail elsewhere [22].

To assist schools with implementing strategies selected as part of the action planning process, intervention schools were provided with the HK toolkit. This comprised: i) An ‘educators’ manual’ which contained booklets on each action area to serve as guides for prioritising action and strategies to address identified priorities; ii) A bin with physical activity resources such as skipping ropes and balls; iii) A resource box with printed materials relating to a healthy lifestyle including the South African Food Based Dietary Guidelines; a poster listing the behaviour outcomes desired for the children; a poster for listing planned actions; and a healthy lifestyle guide for teachers (included in 2011); iv) Nutrition curriculum guidelines integrating the HK goals with the existing Life Orientation curriculum, developed by a curriculum expert in a format familiar to educators.

Principals at schools in the control arm received a booklet with “tips” for healthy schools and a guide to resources that could be accessed to assist in creating a healthier school environment without facilitation from the HK team. After three years the schools were re-assessed in terms of the initial objectives that were developed. In this publication we present results on dietary diversity and snack foods (fat and sugar containing), consumed by children between 2009 and 2011.

### Instruments

Dietary results were obtained by completing an unquantified 24-h recall with each learner in the sample during September to November each year of the intervention period. By restricting the measuring period to the same three months seasonal variation differences were limited. Learners were requested to list all the foods and drinks that they had consumed over a 24-h period starting from the time they awoke until the time they went to bed. Activities of the day were recounted in order to assist them in recalling foods eaten at specific intervals. A dietary diversity score (DDS) was calculated by counting each of nine food groups. The nine groups that were used included: 1) cereals/roots/tubers; 2) meat/poultry/fish; 3) dairy; 4) eggs; 5) vitamin A rich fruit and vegetables; 6) legumes; 7) other fruit; 8) other vegetables; and 9) fats and oils. The food groups were the same as those used in an earlier validation study on children [29]. These were calculated as follows: The South African Food Composition Tables [30] were used to group food items. Each specific food item was included in a group of nine selected food groups as used in the earlier study [29]. A score below four would be indicative of poor dietary diversity (and by association poor food security) while a score of nine would represent a very varied diet. Each food group was only counted once when calculating DDS. The results also included calculating the proportion of people who had consumed a food group at least once.

### Snack consumption

Data from the 24-h recalls were also analysed in terms of snacks consumed per learner with the intention of identifying foods high in fat and sugar and energy-dense. A fat intake score (FIS) was calculated based on seven high fat items which were frequently consumed by learners [1, 22]. These included: fried potato chips; homemade fried foods (fat cakes [fried balls of bread dough], fish cakes, fried chicken), pastries, (pies, sausage rolls and samosas), potato crisps, take-away/fast food, e.g. KFC and McDonalds, processed meats, and other, e.g. gravy, mayonnaise. Similarly, a sugar intake score (SIS) was based on six high sugar items consumed, i.e.: table sugar, chocolates, sweets/candy, confectionary such as cakes/ biscuits/ tarts, jam/syrup, and sugar sweetened beverages. The higher the scores, the higher the intake of fatty and sugary items consumed.

### Statistical analysis

The data was coded and entered into an Excel spreadsheet by an experienced dietitian using the South African Food Composition Tables [30]. Descriptive statistics, namely means and standard deviations were calculated for food items consumed from the fat and sugar groups; consumption of different food groups; number of food groups consumed; and DDS. To evaluate the intervention in this cluster randomised trial an analysis was done at school level since the number of schools were small and we wished to evaluate a broader effect than at individual level only. The mean difference between follow-up (two time points) and baseline was calculated for the primary outcome DDS for each school and a two-sample *t*-test was used to compare the intervention and control arms. The intervention effect was also estimated and reported with 95 % confidence intervals. The observed mean changes within each arm is also reported with 95 % confidence intervals.

### Ethics

Ethical approval for this study was obtained from the Human Research Ethics Committee in the Faculty of Health Sciences, University of Cape Town (HREC REF: 486/2005). In addition, approval for intervention in primary schools was obtained from the Western Cape Education Department. Written informed consent was obtained from the parents of the learners participating in the study.

## Results

The mean age of the children was 9.9 (SD 0.98) in 2009 and 12.3 (SD 0.94) in 2011. In Table 1 findings on snacks, namely, high fat and/or high sugar foods eaten, are presented. It is clear that with one or two exceptions snack food consumption increased from 2009 to 2011. The most commonly eaten fatty food items were potato crisps, followed by processed meat and fried foods. The most commonly consumed sugars were table sugar followed by squashes and then sweets. In the intervention schools there was an increase of more than 10 % in children consuming potato crisps, processed meat, sweets and carbonated beverages between 2009 and 2011. In the control schools there was an increase of more than 10 % in potato crisps, sweets, carbonated beverages and squashes. The intervention effect was not significant for any food items at FU1 or at FU2.

**Table 1:** Percent learners who consumed foods high in fat and/or sugar in 2009, 2010 and 2011 at intervention and control schools based on an unquantified 24-h recall

	Intervention	Control	Intervention effect Mean (95 % CI)	*p*-value
	Mean % (SD)	Mean % (SD)		
Fried potato chips				
Baseline (2009)	6.8 (5.11)	7.4 (4.94)		
FU 1 (2010)	9.5 (5.8)	7.5 (3.69)	2.63 (−4.49; 9.75)	0.442
FU2 (2011)	7.4 (6.57)	6.2 (4.82)	1.80 (−4.21; 7.80)	0.532
Fried food				
Baseline (2009)	25.2 (15.79)	18.9 (8.06)		
FU 1 (2010)	30.1 (17.35)	23.7 (11.56)	0.16 (−14.50; 14.82)	0.982
FU2 (2011)	24.1 (14.91)	21.6 (5.26)	−3.77 (−13.12; 5.58)	0.402
Pies				
Baseline (2009)	3.9 (2.69)	2.1 (1.29)		
FU 1 (2010)	3.1 (3.06)	5.6 (6.91)	−4.31 (−10.16; 1.54)	0.136
FU2 (2011)	2.9 (1.46)	3.4 (6.04)	−2.33 (−7.00; 2.33)	0.302
Potato crisps				
Baseline (2009)	53.1 (14.30)	56.9 (14.38)		
FU 1 (2010)	64.5 (13.46)	70.1 (17.72)	−1.84 (−18.26; 14.58)	0.814
FU2 (2011)	72.3 (9.47	77.1 (8.71)	1.07 (−10.37; 8.22)	0.808
Take away foods				
Baseline (2009)	0.8 (1.66)	0.5 (0.95)		
FU 1 (2010)	1.1 (1.63)	1.1 (1.67)	−0.28 (−2.50; 1.93)	0.788
FU2 (2011)	0.9 (1.26)	1.0 (2.14)	−0.32 (−2.66; 2.03)	0.778
Processed meat				
Baseline (2009)	32.8 (12.75)	35.2 (11.73)		
FU 1 (2010)	42.8 (13.11)	37.7 (14.91)	7.49 (−9.58; 24.56)	0.362
FU2 (2011)	46.7 (15.30)	39.6 (9.74)	9.51 (−2.95; 21.97)	0.124
Table sugar				
Baseline (2009)	80.5 (13.72)	70.8 (17.72		
FU 1 (2010)	86.5 (6.46)	84.1 (11.31)	−7.28 (−20.20; 5.63)	0.247
FU2 (2011)	85.4 (11.28)	81.5 (16.58)	−5.82 (−17.02; 5.39)	0.284
Chocolate				
Baseline (2009)	3.7 (3.96)	4.5 (3.98)		
FU 1 (2010)	2.6 (2.89)	3.2 (3.28)	0.22 (−4.83; 5.28)	0.926
FU2 (2011)	2.0 (1.78)	4.7 (4.68)	−1.81 (−6.79; 3.17)	0.450
Sweets				
Baseline (2009)	26.7 (9.41)	29.9 (10.60)		
FU 1 (2010)	40.9 (15.66)	42.9 (17.90)	1.27 (−19.45; 21.98)	0.898
FU2 (2011)	33.0 (13.28)	43.3 (10.92	−6.96 (−20.14; 6.22)	0.276
Cakes/biscuits				
Baseline (2009)	6.7 (4.94)	5.7 (4.03)		
FU 1 (2010)	5.0 (3.04)	4.7 (5.61)	−0.70 (−5.75; 4.35)	0.770
FU2 (2011)	3.0 (6.93)	6.9 (5.07)	−4.93 (−10.16; 0.31)	0.063
Squashes/cordials				
Baseline (2009)	42.9 (21.46)	45.4 (18.32)		
FU 1 (2010)	44.8 (6.66)	55.2 (17.12)	−8.00 (−27.22; 11.22)	0.387
FU2 (2011)	48.0 (11.89)	55.2 (14.56)	−4.77 (−28.76; 19.21)	0.676
Carbonated beverages				
Baseline (2009)	16 0 (6.65)	10.3 (9.05)		
FU 1 (2010)	25.2 (6.02)	20.5 (11.07)	−0.36 (−11.83; 11.11)	0.947
FU2 (2011)	31.9 (15.52)	26.1 (12.81)	0.80 (−14.37; 16.00)	00.912

*FU1* = follow-up 1 2010; *FU2* = follow-up 2 2011

Table 2 presents data on the diversity of all learners’ diet and how this changed from 2009 to 2011. With the exception of cereals, legumes and nuts and eggs, more learners consumed items from individual food groups in 2011 compared with 2009. All of the learners (100 %) in the intervention group consumed at least one item from the cereal group in 2009, 2010 and in 2011, followed by meat 86.7 to 92.1 %; fats 71.9 to 91.0 % and dairy 70.3 to 75.8 %, in 2009 to 2011, respectively. Intake of eggs was lowest (15.1 to 11.7 %) and for the fruit and vegetable categories. Legumes and nut intake decreased from 53.2 % in 2009 to 39.9 % in 2011. Vitamin A rich fruit and vegetables and fats and oils increased in more than 10 % of children between 2009 and 2011 in both the intervention and control schools while legumes and nuts decreased by more than 10 % of children in this time period. The intervention effect was not significant for any food items at FU1 or at FU2.

**Table 2:** Percent consumption of nine food groups by learners in the intervention and control schools between 2009 and 2011

	Intervention	Control	Intervention effect Mean (95 % CI)	*p*-value
	Mean % (SD)	Mean % (SD)		
Cereals Baseline (2009)	100	100		
FU 1 (2010)	100	100	0	
FU2 (2011)	100	100	0	
Vitamin A rich fruit & veg				
Baseline (2009)	16.0 (5.33)	15.9 (10.83)		
FU 1 (2010)	31.7 (11.62)	26.5 (9.35)	5.1 (−6.78; 17.04)	0.371
FU2 (2011)	30.3 (7.50)	38.1 (6.52)	−7.9 (−19.00:3.18)	0.148
Other fruit				
Baseline (2009)	22.4 (8.67)	21.1 (7.52)		
FU 1 (2010)	30.8 (17.33)	21.4 (16.94)	8.0 (−11.54; 27.50)	0.396
FU2 (2011)	29.1 (11.30)	23.8 (9.83)	3.96 (−8.56; 16.48)	0.508
Other vegetables				
Baseline (2009)	19.3 (8,81)	26.2 (15.8)		
FU 1 (2010)	25.2 (11.20)	26.2 (6.06)	5.84 (−12.24; 23.93)	0.500
FU2 (2011)	25.8 (10.22)	22.6 (6.70)	10.11 (−15.63; 23.08)	0.117
Legumes and nuts				
Baseline (2009)	53.2 (8.61)	55.6 (19.45)		
FU 1 (2010)	48.5 (16.76)	44.3 (23.20)	6.59 (−15.66; 28.83)	0.536
FU2 (2011)	39.9 (15.64)	37.4 (20.81	4.89 (−15.63; 25.40)	0.618
Meat				
Baseline (2009)	86.7 (6.55)	83.1 (9.91)		
FU 1 (2010)	94.6 (4.67)	92.4 (5.54)	−1.37 (−12.24; 9.51)	0.792
FU2 (2011)	92.1 (4.61)	92.1 (5.95)	−3.60 (−14.62; 7.42)	0.495
Fats				
Baseline (2009)	72.0 (11.01)	65.9 (19.53)		
FU 1 (2010)	88.3 (3.88)	82.2 (7.54)	−0.07 (−17.13; 17.00)	0.993
FU2 (2011)	91.0 (5.99)	87.3 (6.75)	−2.42 (−19.75; 14.92)	0.769
Dairy				
Baseline (2009)	70.3 (16.47)	77.2 (14.03)		
FU 1 (2010)	73.5 (8.61)	76.5 (17.38)	3.93 (−11.57; 19.43)	0.595
FU2 (2011)	75.8 (10.89)	76.7 (11.49)	3.02 (−10.27; 16.31)	0.634
Eggs				
Baseline (2009)	15.1 (8.55)	11.4 (7.93)		
FU 1 (2010)	12.8 (5.04)	13.5 (3.66)	−4.40 (−12.69; 3.89)	0.274
FU2 (2011)	11.7 (5.17	12.0 (7.02)	−4.07 (−15.16; 7.02)	0.444

*FU1* = follow-up 1, 2010; *FU2* = follow-up, 2 2011

Most learners in intervention and control groups consumed four or five food groups per day (Table 3). In the intervention schools, 49 % had a DDS ≤4 (poor diversity) in 2009 which increased to 79 % in 2010 and decreased again to 36 % in 2011, showing considerable improvement. In the control schools the percentage DDS ≤ 4 remained fairly similar over this time. In 2011 the intervention schools had considerably less learners with a DDS ≤4 than the control schools.

**Table 3:** Number of food groups eaten by learners at baseline and after the intervention

	Baseline-2009		Follow-up 1-2010		Follow-up 2-2011	
Number of groups eaten	I	C	I	C	I	C
	*N* = 500	*N* = 498	*N* = 522	*N* = 539	*N* = 500	*N* = 543
	No. (%)	No. (%)	No. (%)	No. (%)	No. (%)	No. (%)
1	2 (0.5)	0 (0)	0 (0)	1 (0.3)	1 (0.2)	2 (0.6)
2	12 (2.8)	15 (3.8)	3 (0.8)	7 (1.7)	6 (1.4)	6 (1.7)
3	81 (18.7)	63 (15.8)	37 (9.2)	39 (9.7)	36 (8.5)	38 (10.5)
4	117 (27.0)	117 (29.4)	97 (24.1)	120 (29.7)	108 (25.4)	104 (28.8)
5	124 (28.6)	122 (30.7)	134 (33.3)	142 (35.2)	155 (36.5)	100 (27.7)
6	67 (15.5)	64 (16.1)	83 (20.7)	61 (15.1)	83 (19.5)	73 (20.2)
7	24 (5.5)	12 (3.0)	35 (8.7)	30 (7.4)	29 (6.8)	33 (9.1)
8	5 (1.2)	3 (0.8)	13 (3.2)	4 (1.0)	7 (1.7)	4 (1.1)
9	1 (0.2)	2 (0.5)	0 (0)	0 (0)	0 (0)	0 (0)
Total	433 (100)	396 (100)	402 (100)	404 (100)	424 (100)	360 (100)
DDS ≤4	212 (49 %)	195 (49 %)	317 (79 %)	167 (41 %)	151 (36 %)	150 (42 %)

*I* = intervention schools; *C* = control schools; *DDS* = dietary diversity score

**Fig. 1:**
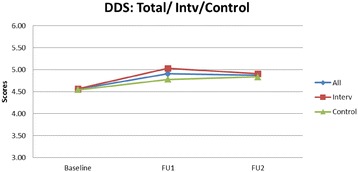
Mean dietary diversity score (DDS) over a three year intervention period

**Fig. 2:**
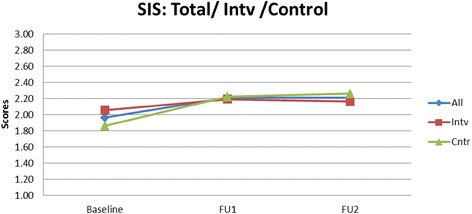
Mean sugar intake score (SIS) over a three year intervention period

**Fig. 3:**
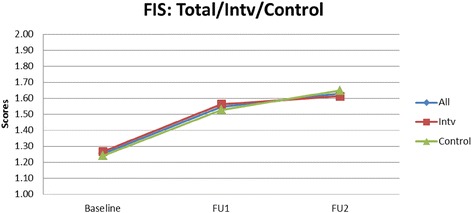
Mean fat intake score (FIS) over a three year intervention period

Table 4 presents findings on the DDS means of the intervention schools increasing from 4.56 to 5.03 to 4.91 (2009, 2010, and 2011). In the control schools the DDS means changed from 4.54 to 4.78 to 4.83. The estimated intervention effects for the primary outcomes are presented. There were no significant intervention effects at any of the two time points for DDS, FIS and SIS. Figures 1, 2, 3 show the mean DDS, FIS and SIS values over the intervention period. Mean FIS increased in both the intervention and control schools over the intervention period, however, the difference between intervention and control groups was not significant at *p* = 0.950 and *p* = 0.809, respectively, neither was the sugar score at *p* = 0.387 and *p* = 0.165 (Table 4).

**Table 4:** The effect of the intervention on dietary diversity score, fat intake score and sugar intake score

Variable		Intervention	Control	Estimated Intervention	*P*-value
				Effect	
DDS		Mean(SD)	Mean(SD)	Mean (95 % CI)	
	Baseline (2009)	4.56(1.29))	4.54(1.22))		
	Follow-up 1 (2010)	5.03(1.24)	4.78(1.17)		
	Follow-up 2 (2011)	4.91(1.17)	4.83(1.29)		
		Mean (95 % CI)	Mean (95 % CI)		
	BSL to FU1	0.49(−0.04;1.03)	0.25(−0.13; 0.62)	0.25 (−0.34; 0.84)	0.387
	BSL to FU2	0.39* (0.10; 0.69)	0.35(−0.01; 0.71))	0.04 (−0.37; 0.46)	0.826
FIS		Mean(SD)	Mean(SD)		
	Baseline (2009)	1.27(0.98)	1.24(0.98)		
	Follow-up 1 (2010)	1.56(0.10)	1.53(0.94)		
	Follow-up 2 (2011)	1.62(0.99)	1.65(0.90)		
		Mean (95 % CI)	Mean (95 % CI)		
	BSL to FU1	0.30 *(0.02; 0.58)	0.29 *(0.11;0.47)	−0.01(−0.29; 0.31)	0.950
	BSL to FU2	0.36 *(0.19; 0.53)	0.38*(0.19;0.57)	−0.03(−0.26; 0.20)	0.809
SIS		Mean(SD)	Mean(SD)		
	Baseline (2009)	2.06(1.07)	1.86(1.11)		
	Follow-up 1 (2010)	2.19(1.09)	2.22(1.12)		
	Follow-up 2 (2011)	2.17(1.06)	2.26(1.22)		
		Mean (95 % CI)	Mean (95 % CI)		
	BSL to FU1	0.13(−0.18;0.44)	0.35(−0.05;0.75)	−0.21(−0.67; 0.25)	0.335
	BSL to FU2	0.11(−0.18;0.40	0.38 *(0.05; 0.72	−0.27 (−0.68; 0.13)	0.165

*DDS* = dietary diversity score; *FIS* = fat intake score; *SIS* = sugar intake score; *CI* = confidence interval; *BSL* = baseline 2009; *FU1* = first follow-up 2010; *FU2* = second follow-up 2011

*Significant improvement over baseline

## Discussion

Findings on dietary diversity in the present study confirm the fact that many schoolchildren from low-income areas have a diet which has little variety as shown by the fact that the majority of children consumed less than four food groups a day. These groups were cereals, meats, fats and oils and dairy. The most neglected food groups were eggs, fruit, vegetables, and legumes. A recent national study on South African children found similar results to these [14]. Furthermore, data on fat and sugar intake of South African adults showed that 18 % had a high fat score and 20 % had a high sugar score [7]. These scores are related to number of foods eaten over a 24 h period. The latter are unfortunately not available in a national sample of South African children.

The children in this study consumed a number of high fat and high sugar foods as indicated by the popular snacks. Crisps, fried foods, sweets, carbonated beverages, to name a few, were commonly consumed at the schools. These items were mostly purchased at the school tuck shops or by vendors selling at the school gates. This is indicative of the fact that the schools do not have policies in place regarding the types of food items for sale on the premises of the school, or that existing policies may not be properly implemented. Since many children are given money to buy food at school [1] it is reasonable to predict that they will purchase items which are cheap and which curb their hunger, Which would often include energy-dense foods that have a low nutritive value [2]. Real progress in promoting healthy eating behaviours can only be attained if effective policies on the sale of foods are implemented and a healthy school environment is promoted [31].

Overall, the intervention did not appear to improve the diet of the learners significantly above that of the control group in terms of dietary diversity and healthier snacking, although there were minor improvements in dietary diversity and restricted intake of sugar. In terms of effect the maintenance of the baseline sugar intake in the intervention schools versus a significant increase in intake in the sugar of controls is the biggest impact of the study. We provide some possible theories for this outcome. Firstly, as shown in data on implementation evaluation, the intervention had poor fidelity [32]. The action planning process was not as successfully undertaken at schools as the research team had hoped. For example, the intervention schools planned to do 30 nutrition events/actions and 27 physical activity events/actions over the three-year period. However, only 20 nutrition and 7 physical activity events were actually undertaken by school staff [32]. Many schools planned events but did not actually carry them out. For example, only two nutrition education activities were undertaken in total at all eight schools over the three year period. In the HK intervention the schools themselves were responsible for implementing the nutrition curriculum guide which they had been provided with. Some of the main reasons given by the teachers for the poor implementation of nutrition events were: being too busy with regular school work and lack of time; poor resources and lack of money; poor physical facilities; poor home environment of many of the learners; lack of parental involvement and unhealthy foods mainly sold at the schools [32].

Secondly, it is important to understand the context in which the intervention took place. In the schools included in the present study issues such as poverty, gangsterism, substance abuse, malnutrition, and diseases associated with infections, e.g. HIV and tuberculosis are still rife in the community [22, 33]. Schools battle with these challenges each day and this could make it difficult for school staff to undertake health promoting activities that are not directly associated with normal day-to-day activities. The issue of NCDs may appear to them as something in the distant future which does not directly affect their everyday lives. Programmes/interventions developed in/settings in developed countries such AS! BC possibly may not face some of the same resource challenges or the social challenges associated with programmes in low-income settings in developing countries.

One of the few studies in South Africa which have evaluated the effect of a nutrition intervention programme on primary school children was undertaken by Oosthuizen et al. [34] in a peri-urban low economic area of the North West Province. Grade 7 children were given nutrition education lessons over a period of one school term. Results were compared with a control group before, immediately after intervention, and nine months later. There was very little dietary variety in the average diet before intervention and minimal changes after intervention; specifically fruit and vegetable intake remained low. The results were disappointing despite the fact that a nutrition programme was developed and implemented by a dedicated person (nutritionist). The researchers attributed this to the fact that children from low-income areas have very little influence on their food choices even though there were improvements in their dietary knowledge. This may also have been a contributory factor in the HK intervention.

In another South African study, Jemmott et al., [35] were able to significantly improve grade 6 adolescents’ consumption, attitudes, and knowledge of fruit and vegetables in a yearlong school intervention undertaken in a low-income urban community of the Eastern Cape. However, their intervention approach differed completely. Instead of relying on the schools to plan and execute their own activities, they used trained facilitators from outside the schools to implement 12 modules which comprised interactive exercises, games, brainstorming, role-playing and group discussions. At follow-up participants in the intervention ate about 0.54 (*p* = 0.003) more servings of fruit per day and 0.77 (*p* = 0.0001) more vegetables compared to participants who did not receive the intervention. Clearly a programme which utilised expertise from outside the schools appeared to have a greater success at improving the quality of primary school children’s diet. However, the sustainability and cost of such an intervention would have to be weighed against its long-term successes.

A number of studies undertaken in other countries have shown that using trained facilitators to implement a nutrition curriculum in primary schools resulted in successful outcomes in children’s knowledge and eating behaviour [36, 37]. The main difference between these studies and the present one appears to reside in the fact that teachers in the HK study were provided with nutrition guidelines to be integrated into the overall Life Orientation curriculum as part of the action planning process to render it more sustainable. However, *implementation* of the curriculum guidelines was their own choice. In the other studies referred to [35, 36], nutrition educators from outside the school or educators at the schools were trained to follow the nutrition curriculum in a set (compulsory) manner.

We recognise that one of the limitations of the study was the dietary method used, namely a single 24-h recall which relied on learners remembering what they had eaten the day before. The results may hence appear biased in terms of food items that were left out. However coding of the data was done by the same experienced dietitian at all the time intervals. Furthermore, the fact that the 24 h recall was unquantified means that no distinction is made in whether children ate small or large portions of food items.

### Recommendations

Lessons learnt from the current study imply that learners’ eating behaviours are unlikely to improve if they are exposed to a school environment that does not specifically promote healthy behaviours. Furthermore, the Department of Basic Education needs to consider ways of strengthening the implementation of the existing ‘Integrated School Health Policy’ to ensure healthy nutrition and physical activity environments in schools in all socio-economic settings.

## Conclusions

The results of the HK intervention were disappointing in terms of improvement in the variety of the diet and a decrease in unhealthy snacking. We attribute this to the finding that our model using action planning by the schools may not have been the best model for a setting where the majority of children came from low-income communities. Schools appeared to have difficulty in actually following through with the action plans that they developed based on their own assessment of their school contexts. Furthermore, schools did not appear to address the issue of promoting and implementing a healthy environment and little was available in terms of school policies that aimed at fostering a healthy lifestyle.
